# Diverse Mentoring Connections Across Institutional Boundaries in the Biomedical Sciences: Innovative Graph Database Analysis

**DOI:** 10.2196/47560

**Published:** 2024-06-17

**Authors:** Toufeeq Ahmed Syed, Erika L Thompson, Zainab Latif, Jay Johnson, Damaris Javier, Katie Stinson, Gabrielle Saleh, Jamboor K Vishwanatha

**Affiliations:** 1 University of Texas Health Science Center at Houston Houston, TX United States; 2 University of Texas Health Science Center at San Antonio San Antonio, TX United States; 3 Northwestern University Evanston, IL United States; 4 University of North Texas Health Science Center at Fort Worth Fort Worth, TX United States

**Keywords:** online platform, mentorship, diversity, network analysis, graph database, online communities

## Abstract

**Background:**

With an overarching goal of increasing diversity and inclusion in biomedical sciences, the National Research Mentoring Network (NRMN) developed a web-based national mentoring platform (MyNRMN) that seeks to connect mentors and mentees to support the persistence of underrepresented minorities in the biomedical sciences. As of May 15, 2024, the MyNRMN platform, which provides mentoring, networking, and professional development tools, has facilitated more than 12,100 unique mentoring connections between faculty, students, and researchers in the biomedical domain.

**Objective:**

This study aimed to examine the large-scale mentoring connections facilitated by our web-based platform between students (mentees) and faculty (mentors) across institutional and geographic boundaries. Using an innovative graph database, we analyzed diverse mentoring connections between mentors and mentees across demographic characteristics in the biomedical sciences.

**Methods:**

Through the MyNRMN platform, we observed profile data and analyzed mentoring connections made between students and faculty across institutional boundaries by race, ethnicity, gender, institution type, and educational attainment between July 1, 2016, and May 31, 2021.

**Results:**

In total, there were 15,024 connections with 2222 mentees and 1652 mentors across 1625 institutions contributing data. Female mentees participated in the highest number of connections (3996/6108, 65%), whereas female mentors participated in 58% (5206/8916) of the connections. Black mentees made up 38% (2297/6108) of the connections, whereas White mentors participated in 56% (5036/8916) of the connections. Mentees were predominately from institutions classified as Research 1 (R1; doctoral universities—very high research activity) and historically Black colleges and universities (556/2222, 25% and 307/2222, 14%, respectively), whereas 31% (504/1652) of mentors were from R1 institutions.

**Conclusions:**

To date, the utility of mentoring connections across institutions throughout the United States and how mentors and mentees are connected is unknown. This study examined these connections and the diversity of these connections using an extensive web-based mentoring network.

## Introduction

Web-based networks have the power and capacity to connect individuals unlike anything we have previously experienced in our society. This connection capacity is especially important for those traditionally underrepresented in the biomedical sciences. Black or African American and Hispanic or Latinx individuals use social media at higher rates than their White counterparts, which may aid their ability to seek mentoring beyond their local networks [1]. This is especially important in the realm of health care as diversity in the biomedical workforce is essential for addressing health disparities and other public health needs [2-4]. The National Institutes of Health (NIH) has invested in diversity-focused initiatives to promote representation from underrepresented groups in higher levels of the biomedical workforce [3]. One of these investments is in the National Research Mentoring Network (NRMN), which aims to promote diversity among undergraduate, graduate, postdoctoral, and workforce career stages through mentorship and mentoring tools [5,6].

NRMN provides resources (eg, culturally responsive mentoring and networking) remotely through a portal, NRMNet, for the biomedical, educational, and workforce pipeline [5]. A key component of NRMNet is the MyNRMN platform, which allows people from all education and career stages to remotely mentor or receive mentorship throughout the United States and territories [7]. Mentoring is an integral factor in supporting longevity and persistence in the biomedical science fields and training, as well as for professional development and developing a science identity [8-11]. Moreover, mentoring early and during impressionable stages in a person’s education and career pathways may have a cumulative effect for downstream biomedical career success [12]. By engaging with MyNRMN, users gain access to a network of mentors and mentees promoting education and career advancement for individuals who may not have access to these resources locally.

Historically, persons from underrepresented minority groups are at a disadvantage when accessing and receiving mentoring compared with nonminority groups [13]. Mentorship from persons who identify with challenges specific to underrepresented minorities is a valuable asset for professional development and training [14]. Off-site mentorship and self-reflection are key aspects to influential and beneficial mentorships, which often are not accessible within limited networks [15]. Furthermore, mentoring or coaching networks, including peer mentors, provide different forms of support for training and development [12,15,16].

MyNRMN provides an accessible platform for mentorship with the goal of providing meaningful connections for persistence in the biomedical sciences. The MyNRMN platform is unique because it crosses state lines and institutional boundaries for mentorship, which is vital for underrepresented minorities who may not have access to mentoring networks or expansive social capital at their own institution. The development of MyNRMN, as discussed by Ahmed et al [7], describes the importance of incorporating social capital and social networks while building and creating this platform. The connections built within MyNRMN increase an individual’s social capital, specifically in the biomedical sciences, enabling them to move further in their education and career through the “informational, emotional, and instrumental resources and supports” [7]. By observing and understanding the connections within MyNRMN, we can observe how an individual’s social capital increases through this network. Future studies will discuss the effects of increased social capital within MyNRMN.

This study aimed to examine the large-scale mentoring connections, facilitated by MyNRMN, between students (mentees) and faculty (mentors) across institutional and geographic boundaries on a large, national mentoring network for biomedical sciences. We assessed the diversity of connections on the platform by gender, race, ethnicity, educational attainment, and minority-serving institution (MSI)–Carnegie classification. We hypothesized that building this web-based platform would create a space for diverse mentoring networks that expand beyond an individual’s immediate proximity and personal identity.

## Methods

### MyNRMN Platform

Details of the MyNRMN platform capabilities have been described in previous publications [5,7]. Briefly, the platform is a remote mentoring, networking, and career development space where individuals can enroll, create a profile, and sign up to be a mentor or mentee within the network. Additionally, the platform provides a space for “connections” (ie, people linking accounts across the platform) and to form mentoring relationships. In typical academic settings, mentoring is limited to a particular department or institution, but with MyNRMN, a mentee or mentor can connect with a plethora of like-minded biomedical science professionals and students across the nation (8473 mentors and 15,852 mentees as of May 15, 2024), increasing their social capital beyond their immediate location. This includes connections across types of institutions and professional or education levels. For example, a mentee from a 2-year community college in Idaho can connect with a mentor from an institution classified by Carnegie as a Research 1 (R1; doctoral universities—very high research activity) on the East Coast, or a mentee from a 4-year program in Atlanta can connect with somebody from Washington state in a PhD program. In this fashion, MyNRMN can facilitate interactions for those seeking professional or career support, or advice on majors, job opportunities, research areas, graduate school applications, and so on.

### Graph Database Infrastructure

**Figure 1:**
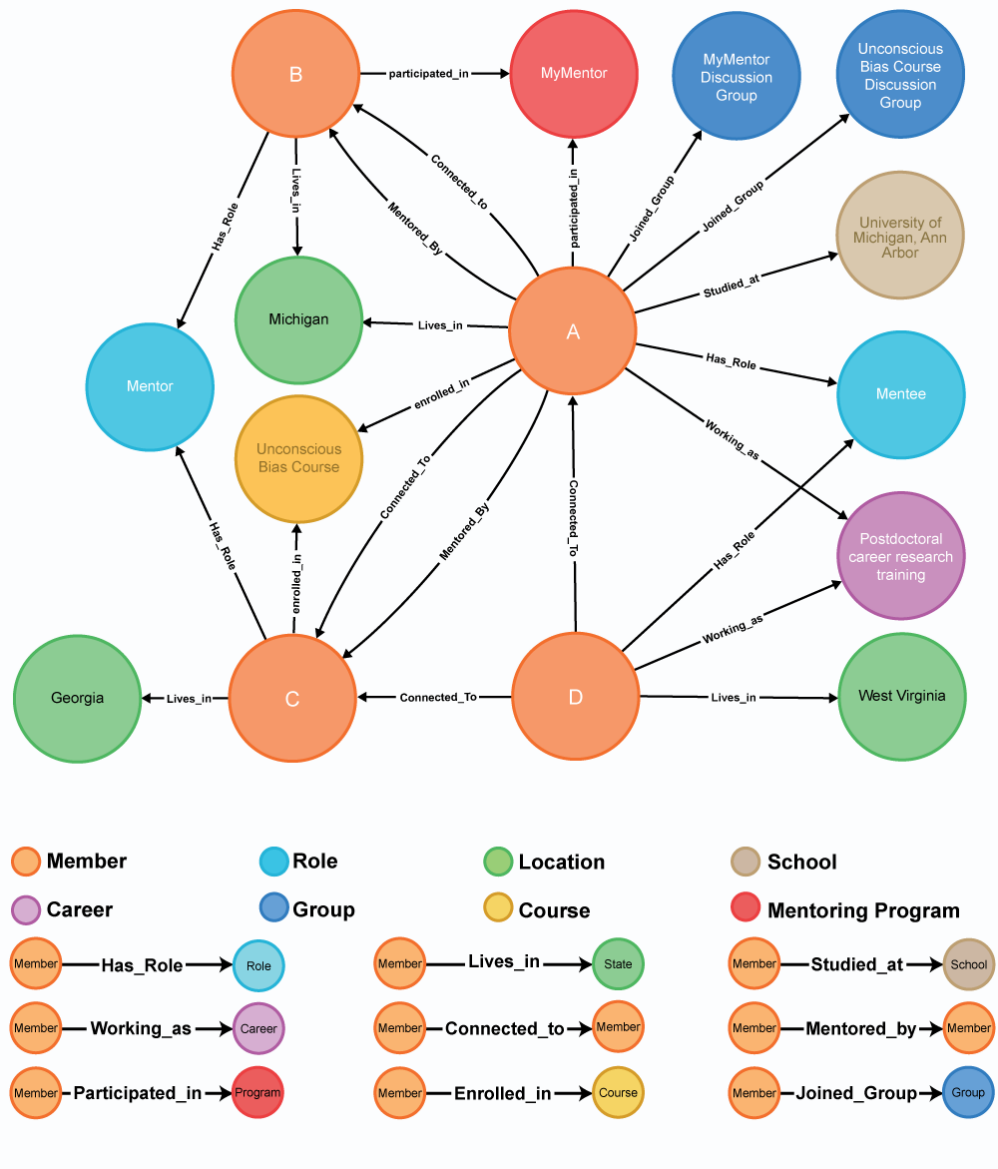
MyNRMN Neo4j graph database structure.

MyNRMN is built on a robust graph database called Neo4j, which facilitates creating a rich social graph that mimics real-world social and mentoring connections. Each node can be a mentee, mentor, member, institution, career, location, group, course, mentoring program, or cohort. Additionally, each connection (the link between 2 nodes) is categorized and labeled. For example, a link between a mentor and a mentee is labeled as a mentor-mentee connection and a link between a mentee and a mentee is labeled as a mentee-mentee connection. Figure 1 explains the different types of nodes and the labels of links between the nodes. We have built our platform and graph database to work together, so that every time a mentee connects to a mentor, a mentor connects to another mentor, a mentee connects to another mentee, or a member joins a group, the system creates an entry in our database and captures that in the graph database as a connection by these 2 nodes.

### Measures

In the MyNRMN platform, we capture the profile information, which includes demographic fields (eg, gender, race, ethnicity, institution, whether they are first in the family to go to college, and education level). When a member joins MyNRMN, the system syncs the member’s profile to our Neo4j graph database, creating a node for the person, location, school, and career, and creates the links between these nodes. Each node includes properties such as system ID and name. For example, the member node includes the member’s profile information, for example, system ID, full name, email, alternate email, education, degrees, careers, interest, and has changed school in past 6 months. Likewise, each activity in MyNRMN is also recorded in Neo4j. Through this process, when a member joins a group, enrolls into a course, or joins a cohort, a link is created between the member node and the node of the group, the course, or the cohort the member has joined.

For member connections, the system only records accepted connections; pending and rejected connection requests are not posted to our graph database. However, connections can be made in a multitude of ways using different engagement features [7]. For example, a mentee can start a search or seek a mentor using the Find a Mentor tool. The mentee will start with a few keywords and search for different mentors. Once they find a mentor they are interested in connecting with and seeking advice from, they will send a request for connection. The mentor will receive a connection request via email and will have the choice to accept or reject that request. If the request is accepted, the system will create a Connected_to link between the mentee and the mentor nodes in Neo4j. If the request is of a formal mentoring type, a Mentored_by link will be created between the mentee and the mentor nodes. The link can also include properties just like nodes; for example, the Mentored_by link includes information such as mentorship type (one-on-one mentoring or program mentoring), program name, and mentoring path (postbaccalaureate, undergraduate, graduate, postdoctoral, and junior faculty).

If the connection is not accepted, it will stay pending in our platform and will not be added to our graph database. Hence, our graph database maintains the active connections between mentors and mentees. For this paper, we are reporting the network and connection analysis between mentors and mentees for active, accepted connections. To describe our data set, we have extracted 3 different types of data sets analyzing mentee-mentor connections, mentee-mentee connections, and mentor-mentor connections (the latter 2 are considered peer mentoring). For each of these analyses, we have included only the accepted connections on our platform.

For each variable, such as gender, race, ethnicity, education level, and MSI-Carnegie classification, we counted the number of accepted connections based on the role of the requester (member who requested the connection) and the role of the receiver (member who accepted the connection) as follows: mentee-mentor, mentee-mentee, and mentor-mentor. To obtain the number of connections between the categories defined above, we retrieved the profile information of the requester and the receiver of the connection request. For gender, race, ethnicity, and education level, we used the information provided by the member in their profile. The connections by MSI-Carnegie institutions were determined by the institution stated in the member profile. These were then classified into one of the following categories: Asian American and Native American Pacific Islander–serving institutions (AANAPISIs), historically Black colleges and universities (HBCUs), Hispanic-serving institutions (HSIs), tribally controlled colleges and universities (TCCUs), Carnegie doctoral university R1, and Carnegie doctoral university R2 (doctoral universities—high research activity). We have defined the MSIs based on the US Department of Education (2020) Eligibility Matrix Report. The Carnegie classification of institutions was based on The Carnegie Classification of Institutions of Higher Education [17].

### Sample

To examine the mentoring connections occurring on MyNRMN, we captured data from July 1, 2016, until May 31, 2021. These data show the MyNRMN connections between mentees and mentors from different genders, races, ethnicities, education levels, and MSI-Carnegie institutions across the country, based on the user’s profile data. During this time frame there were 2261 mentees and 1583 mentors contributing connection data.

### Data Analysis

This study presents the descriptive counts and frequencies for connections accepted across the MyNRMN platform. These connections are stratified by demographics and institution type. Moreover, we estimated the average connections by demographic and institution characteristics. Alluvial graphs were developed to show the percentage of accepted connections between platform users.

### Ethical Considerations

Research reported in this publication was supported by the National Institute of General Medical Sciences of the NIH under award numbers U54GM119023 and U24GM132217. The content is solely the responsibility of the authors and does not necessarily represent the official views of the NIH. All user data collected is protected under the North Texas Regional Institutional Review Board, reference number 2015-0720, and stored securely.

## Results

### Characteristics

During the July 1, 2016, to May 31, 2021, time frame, there were 2222 mentees and 1652 mentors contributing connection data. Tables 1 and 2 describe the number of connections and average connections per user during the study period stratified by gender, race, ethnicity, and institution type. In total, there were 15,024 connections during the study period. Among the connections collected in the aforementioned window, mentors and mentees came from 1625 institutions. The institutions with the highest representation included the partner institutions Tuskegee University (151 connections), Savannah State University (98 connections), University of North Texas Health Science Center (97 connections), and University of Wisconsin-Madison (93 connections). A majority of connections occurred between different institutions (n=4603 connections).

**Table 1:** Mentor and mentee connections.

Demographic		Mentees, n^a^	Connections by mentees, n^b^	Connections per mentee, mean (SD)	Mentors, n^c^	Connections by mentors, n^d^	Connections per mentor, mean (SD)
Total		2222	6108	2.75 (5.39)	1652	8916	5.54 (16.52)
**Gender**							
	Female	1309	3996	3.05 (6.11)	895	5206	5.82 (19.73)
	Male	499	1345	2.7 (5.11)	624	3217	5.16 (12.42)
	Other	9	27	3 (3.84)	4	9	2.25 (1.5)
**Race**							
	White	638	1590	2.49 (4.4)	902	5036	5.58 (19.63)
	Black	727	2297	3.16 (4.97)	277	1592	5.75 (10.92)
	Asian	281	603	2.15 (3.05)	178	902	5.07 (17.83)
	Native American	52	95	1.83 (1.54)	16	51	3.19 (3.15)
	Hawaiian-Pacific Islander	11	14	1.27 (0.65)	6	9	1.5 (0.84)
	Two or more races	20	121	6.05 (15.88)	19	115	6.05 (5.25)
	Other	124	314	2.53 (3.65)	49	341	6.96 (10.99)
**Ethnicity**							
	Hispanic or Latino	349	943	2.7 (4.5)	172	908	5.28 (8.71)
	Not Latino	1208	3681	3.05 (6.38)	1169	6210	5.31 (13)
	Other	85	237	2.79 (4.62)	51	268	5.25 (8.66)
**Education**							
	Undergraduate	563	1703	3.02 (5.87)	33	239	7.24 (16.07)
	Graduate	470	1440	3.06 (5.27)	77	868	11.27 (54.55)
	Postdoctoral	292	716	2.45 (3.08)	205	885	4.32 (6.52)
	Other (currently working/faculty/not in school or formal program)	437	1378	3.15 (7.82)	1221	6574	5.38 (12.89)
**Institution**							
	AANAPISI^e^	48	155	3.23 (5.3)	25	151	6.04 (7.97)
	HBCU^f^	307	1072	3.49 (5.87)	121	684	5.65 (6.54)
	HSI^g^	284	538	1.89 (1.89)	105	560	5.33 (12.76)

^a^Number of mentees with at least 1 active connection.

^b^Number of active connections requested or accepted by mentees.

^c^Number of mentors with at least 1 active connection.

^d^Number of active connections requested or accepted by mentors.

^e^AANAPISI: Asian American and Native American Pacific Islander–serving institution.

^f^HBCU: historically Black colleges and universities.

^g^HSI: Hispanic-serving institution.

**Table 2:** Connection numbers by type of connection.

			Mentee- mentor connections, n^a^		Mentee- mentor connections, mean (SD)		Mentor- mentee connections, n^b^		Mentor- mentee connections, mean (SD)		Mentee- mentee connections, n^c^		Mentee- mentee connections, mean (SD)		Mentor- mentor connections, n^d^		Mentor- mentor connections, mean (SD)
Total			3388		2.17 (4.25)		930		2.93 (9.44)		895		2.05 (2.79)		2299		4.35 (14)
**Gender**																	
	Female	2125		2.37 (4.47)		500		2.92 (11.14)		636		2.42 (3.3)		1441		5.06 (17.08)	
	Male	769		2.43 (5.44)		357		3.16 (7.84)		150		1.58 (1.37)		719		3.84 (10.27)	
	Other	9		1.13 (0.35)		0		0 (0.0)		2		1 (0.0)		0		0 (0.0)	
**Race**																	
	White	841		2.02 (2.68)		572		3.4 (12.77)		226		2.09 (3.45)		1295		4.89 (17.39)	
	Black	1166		2.24 (4.03)		161		2.6 (2.46)		405		2.29 (2.49)		367		3.67 (6.1)	
	Asian	392		1.95 (2.87)		79		3.16 (3.65)		69		1.44 (0.85)		295		5.67 (18.47)	
	Native American	36		1.29 (0.53)		9		2.25 (1.5)		5		1 (0.0)		16		2 (2.07)	
	Hawaiian-Pacific Islander	10		1.25 (0.71)		1		1 (0.0)		0		0 (0.0)		4		1.33 (0.58)	
	Two or more races	96		6.4 (17.13)		11		3.67 (2.08)		5		1 (0.0)		4		1.33 (0.58)	
	Other	190		1.98 (2.64)		27		2.08 (1.71)		36		2.12 (2.18)		105		5 (8.14)	
**Ethnicity**																	
	Hispanic or Latino	530		2.29 (4.73)		61		1.97 (1.68)		109		1.68 (1.15)		153		3.4 (4.14)	
	Not Latino	1927		2.41 (4.86)		581		2.7 (5.9)		583		2.39 (3.44)		1487		4.14 (9.11)	
	Other	146		2.56 (3.7)		21		2.1 (2.02)		23		2.09 (2.7)		82		4.82 (6.54)	
**Education**																	
	Undergraduate	916		2.25 (5.58)		118		8.43 (21.02)		323		2.15 (2.18)		442		3.86 (3.89)	
	Graduate	890		2.51 (3.88)		193		9.19 (31.17)		192		2.31 (3.69)		1499		12.63 (45.62)	
	Postdoctoral	369		2.32 (2.92)		62		1.77 (2.14)		68		1.33 (1.07)		189		3.32 (3.01)	
	Other (currently working/faculty/not in school or formal program)	648		2.58 (5.58)		495		2.31 (21.02)		184		2.75 (4.23)		81		4.12 (8.94)	
**Institution**																	
	AANAPISI^e^	106		3.79 (6.56)		14		1.56 (1.01)		9		1.8 (0.84)		29		2.9 (3.51)	
	HBCU^f^	463		2.19 (5.18)		105		2.69 (2.76)		246		2.37 (2.24)		143		2.8 (3.21)	
	HSI^g^	311		1.47 (1.3)		27		2.08 (1.89)		60		1.46 (0.74)		106		3.21 (4.28)	

^a^Number of connections requested by mentees and accepted by mentors.

^b^Number of connections requested by mentors and accepted by mentees.

^c^Number of connections requested by mentees and accepted by other mentees.

^d^Number of connections requested by mentors and accepted by other mentors.

^e^AANAPISI: Asian American and Native American Pacific Islander–serving institution.

^f^HBCU: historically Black colleges and universities.

^g^HSI: Hispanic-serving institution.

### Gender

#### Mentee-Mentor Accepted Connections by Gender

**Figure 2:**
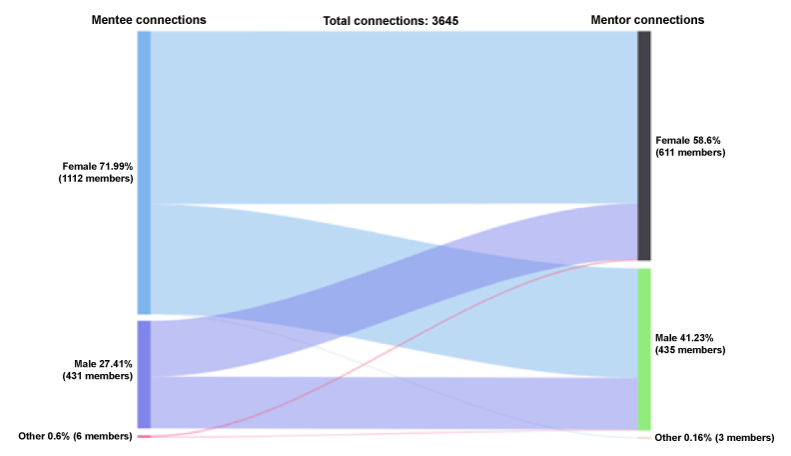
Connections by gender: mentee-mentor.

Of the accepted connections between mentees and mentors (n=3645 connections), female mentees made up 72% (2624/3645) of the accepted connections, male mentees made up 27% (999/3645), and those who identified as other gender made up 0.6% (22/3645); see Figure 2. Alternatively, female mentors made up 59% (2136/3645) of the accepted connections, male mentors made up 41% (1503/3645), and those who identified as other made up 0.2% (6/3645) of the accepted connections. Of the accepted connections, female mentees connected with female mentors approximately 61% (1604/2624) of the time, and they connected with male mentors approximately 39% (1016/2624) of the time. Male mentees were almost evenly split in their connection percentages between male and female mentors. Of the accepted connections, female mentors connected with female mentees approximately 75% (1604/2136) of the time, whereas male mentors connected with female mentees approximately 68% (1016/1503) of the time.

#### Mentee-Mentee Accepted Connections by Gender

**Figure 3:**
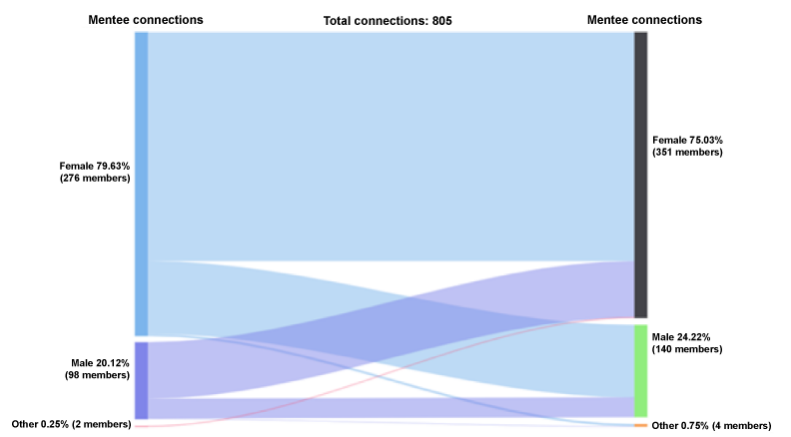
Connections by gender: mentee-mentee.

Female mentees represented 80% (641/805) of the initiated and accepted mentee-mentee connections (n=805 connections), whereas male mentees made up 20% (162/805) and those who identify as other gender represented 0.25% (2/805); see Figure 3. Among the mentees who received and accepted peer mentee requests, 75% (604/805) were female, 24% (195/805) were male, and 0.75% (6/805) identified as other gender. Female mentees who initiated the mentee-mentee connection reached out to female mentees 75% (483/641) of the time, male mentees 24% (153/641) of the time, and those who identified as other 0.8% (5/641) of the time. Alternatively, male mentees who initiated the mentee-mentee connection reached out to female mentees 73% (119/162) of the time, male mentees 26% (42/162) of the time, and those who identified as other 0.6% (1/162) of the time. The mentees who identified as other connected only with female mentees.

#### Mentor-Mentor Accepted Connections by Gender

**Figure 4:**
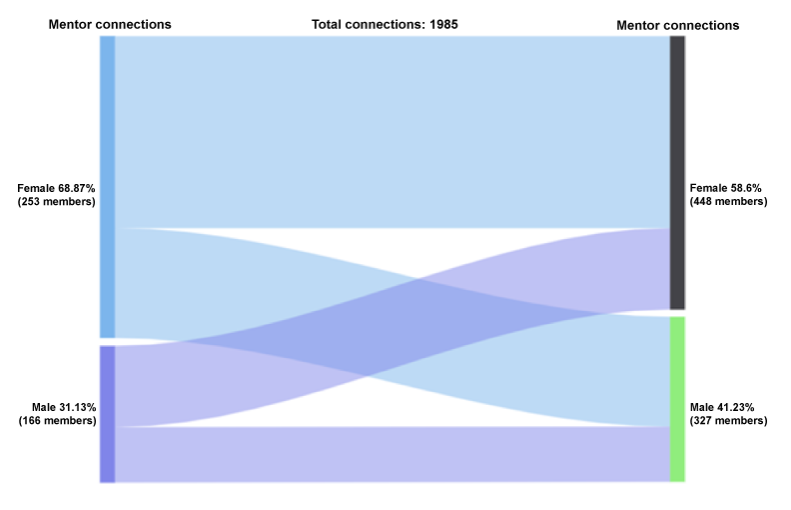
Connections by gender: mentor-mentor.

Mentors can also connect with one another. Of the mentors who initiated the accepted mentoring connections (n=1985 connections), 69% (1367/1985) were female and 31% (618/1985) were male (Figure 4). Among the mentors who received and accepted peer mentor requests, 62% (1237/1985) were female and 38% (748/1985) were male. Among the female-initiated mentor-mentor connections, 64% (869/1367) were female-female and 36% (498/1367) were female-male connections. Among the male-initiated mentor-mentor connections, 60% (368/618) were male-female and 40% (250/618) were male-male connections.

### Race

#### Mentee-Mentor Accepted Connections by Race

**Figure 5:**
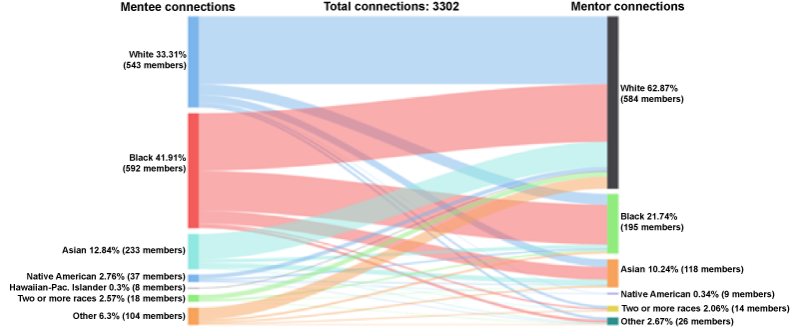
Connections by race: mentee-mentor.

Of the accepted connections between mentees and mentors (n=3302 connections), mentee connections were 42% (1384/3302) Black, 33% (1100/3302) White, 13% (424/3302) Asian, 3% (91/3302) Native American, 3% (85/3302) 2 or more races, 0.3% (10/3302) Hawaiian-Pacific Islander, and 6% (208/3302) other (Figure 5). Alternatively, 63% (2,076/3302) of accepted mentor connections were White mentors, followed by 22% (718/3302) Black, 10% (338/3302) Asian, 2% (68/3302) 2 or more races, 0.4% (12/3302) Native American, and 3% (88/3302) other.

Of the 1100 accepted connections among White mentees, the mentors were 74% (817/1100) White, 12% (131/1100) Black, 8% (90/1100) Asian, 0.3% (3/1100) Native American, 3% (28/1100) 2 or more races, and 3% (31/1100) other. Of the 1384 accepted connections among Black mentees, the mentors were 50% (694/1384) White, 35% (481/1384) Black, 11% (155/1384) Asian, 0.1% (1/1384) Native American, 0.1% (1/1384) Hawaiian-Pacific Islander, 1% (19/1384) 2 or more races, and 3% (33/1384) other. Among the 424 accepted connections among Asian mentees, the mentors were 72% (306/424) White, 10% (43/424) Black, 14% (59/424) Asian, 0.2% (1/424) Native American, 1% (6/424) 2 or more races, and 2% (9/424) other. Among the 91 accepted connections among Native American mentees, the mentors were 55% (50/91) White, 21% (19/91) Black, 10% (9/91) Asian, 7% (6/91) Native American, 0% (0/91) Hawaiian-Pacific Islander, 1% (1/91) 2 or more races, and 7% (6/91) other. Among the 85 connections for mentees identifying as 2 or more races, mentors were 68% (58/85) White, 22% (19/85) Black, 7% (6/85) Asian, and 2% (2/85) other. Among the 10 connections for Hawaiian-Pacific Islander mentees, mentors were 70% (7/10) White, 10% (1/10) Black, 10% (1/10) Hawaiian-Pacific Islander, and 10% (1/10) 2 or more races. Finally, among the 208 connections from mentees identifying as other, mentors were 69% (144/208) White, 12% (24/208) Black, 9% (19/208) Asian, 0.5% (1/208) Native American, 6% (13/208) 2 or more races, and 4% (7/208) other.

Of the 2076 connections accepted by White mentors, mentees were 39% (817/2076) White, 33% (694/2076) Black, 15% (306/2076) Asian, 2% (50/2076) Native American, 0.3% (7/2076) Hawaiian-Pacific Islander, 3% (58/2076) 2 or more races, and 7% (144/2076) other. Of the 718 connections accepted by Black mentors, mentees were 18% (131/718) White, 67% (481/718) Black, 6% (43/718) Asian, 3% (19/718) Native American, 0.1% (1/718) Hawaiian-Pacific Islander, 3% (19/418) 2 or more races, and 3% (24/718) other. Among the 338 connections accepted by Asian mentors, mentees were majority Black 46% (155/338), followed by White (90/338, 27%), Asian (59/338, 17%), other race (19/338, 6%), Native American (9/338, 3%), and 2 or more races (6/338, 2%). Of the 88 connections accepted by mentors who identified as other race, approximately one-third were with Black and White mentees (33/88, 39% and 31/88, 35%, respectively). The remaining accepted connections comprised Asian (9/88, 10%), other race (7/88, 8%), Native American (6/88, 7%), and mentees of 2 or more races (2/88, 2%). Among the 68 connections accepted by mentors of 2 or more races, a majority were with White mentees (28/68, 41%), followed by Black (19/68, 28%), other (13/68, 19%), Asian (6/68, 9%), Native American (1/68, 1%), and Hawaiian-Pacific Islander (1/68, 1%). Only 12 connections were accepted by Native American mentors, and half were with Native American mentees. Similarly, there were only 2 connections accepted by Hawaiian-Pacific Islander mentors.

#### Mentee-Mentee Accepted Connections by Race

**Figure 6:**
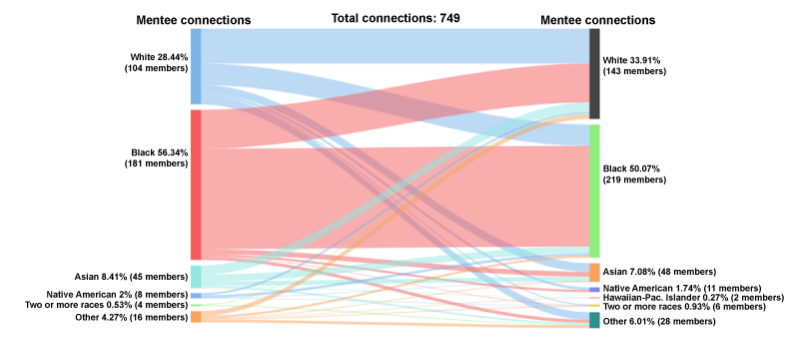
Connections by race: mentee-mentee.

Black mentees represented 56% (422/749) of the initiated and accepted mentee-mentee connections (n=749 connections), followed by 28% (213/749) White, 8% (63/749) Asian, 2% (15/749) Native American, 4% (32/749) other, and 0.5% (4/749) 2 or more races (Figure 6). Among the mentees who received and accepted peer mentee requests, 50% (375/749) were Black, 34% (254/749) were White, 7% (53/749) were Asian, 2% (13/749) were Native American, 0.3% (2/749) were Hawaiian-Pacific Islander, 1% (7/749) were 2 or more races, and 6% (45/749) identified as other race.

Black mentees initiated the most connections compared with other racial groups. Black mentees who initiated peer mentee-mentee connections (n=422 connections) connected with mentees who were Black (283/422, 67%), White (109/422, 26%), and less than 3% of Asian (14/422), Native American (6/422), other (9/422), and 2 or more races (1/422). White mentees who initiated the mentee-mentee connections (n=213 connections) reached out to mentees who were White (98/213, 46%), Black (60/213, 28%), Asian (24/213, 11%), Native American (7/213, 3%), other race (21/213, 10%), and 2 or more races (3/213, 1%). Asian mentees initiated 63 connections. These connections were directed toward mentees who were White (26/63, 41%), Black (19/63, 30%), Asian (12/63, 19%), other race (5/63, 8%), and Hawaiian-Pacific Islander (1/63, 2%). Mentees of other racial backgrounds initiated 32 connections primarily to White (13/32, 41%), other (8/32, 25%), and Black (19%, 6/32) mentees. The remaining connections were with Asian mentees (2/32, 6%), mentees of 2 or more races (2/32, 6%), and Hawaiian-Pacific Islander (1/32, 3%) mentees. A total of 15 mentee-mentee connections were initiated among Native American mentees; of these connections, 7/15 (47%) were with Black mentees, 6/15 (40%) to White mentees, 1/15 to Asian mentees, and 1/15 to mentees of 2 or more races. Only 4 connections were initiated by mentees of 2 or more races.

#### Mentor-Mentor Accepted Connections by Race

**Figure 7:**
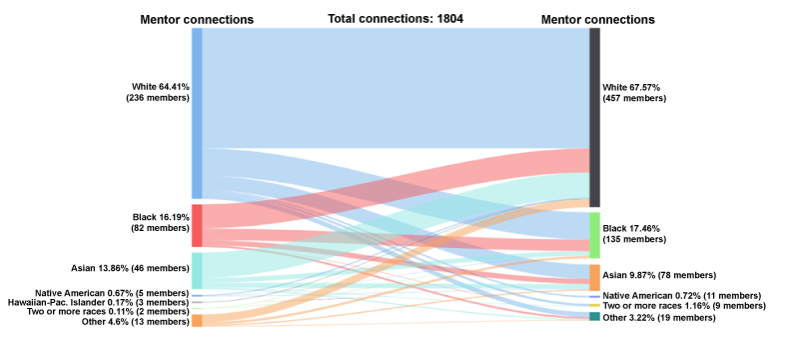
Connections by race: mentor-mentor.

Of the mentors who initiated the accepted mentoring connections (n=1804 connections), 64% (1162/1804) were White, 16% (292/1804) were Black, 14% (250/1804) were Asian, 5% (83/1804) were other race, and less than 1% were Native American (12/1804), Hawaiian-Pacific Islander (3/1804), and 2 or more races (2/1804); see Figure 7. Among the mentors who received and accepted peer mentor requests, 68% (1219/1804) were White, 17% (315/1804) were Black, 10% (178/1804) were Asian, 3% (58/1804) were other, 1% (21/1804) were 2 or more races, and 0.7% (13/1804) were Native American.

White mentors had the most initiated connections compared with other racial groups. White mentors who initiated peer mentor-mentor connections (n=1162 connections) connected with White (818/1162, 70%), Black (186/1162, 16%), and Asian (98/1162, 8%) mentors. The remaining connections comprised less than 5% (Native American, 2 or more races, and other combined total 60/1162) of the initiated connections. Black mentors who initiated mentor-mentor connections (n=292 connections) reached out to White (164/292, 56%), Black (79/292, 27%), Asian (35/292, 12%), and other race (13/292, 5%) mentors. Asian mentors initiated 250 connections to White (172/250, 69%), Black (32/292, 13%), and Asian (33/250, 13%) mentors. The remaining connections comprised less than 5% (Native American, 2 or more races, and other combined total 13/250) of initiated connections. Mentors of other racial backgrounds initiated 83 connections primarily to White (54/83, 95%), Black (16/83, 19%), and Asian (9/83, 11%) mentors. The remaining 5% (4/83) of connections were initiated by mentors of other racial backgrounds. Twelve mentor-mentor connections were initiated among Native American mentors; of these connections, 67% (8/12) were with White mentors. Only 5 total connections were initiated by mentors from Hawaiian-Pacific Islander and more than 1 racial background.

### Ethnicity

#### Mentee-Mentor Accepted Connections by Ethnicity

**Figure 8:**
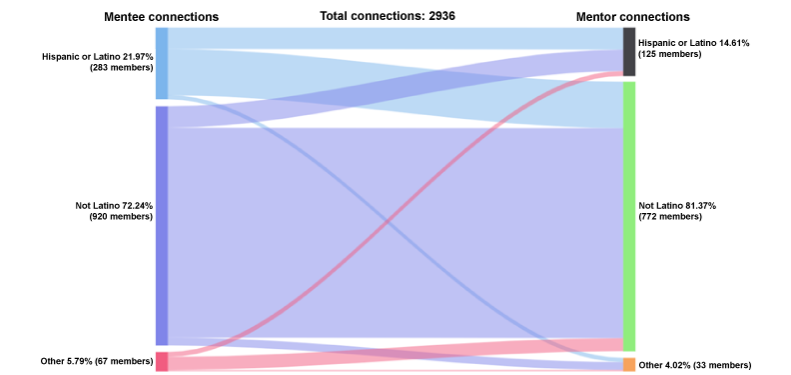
Connections by ethnicity: mentee-mentor.

Of the accepted connections between mentees and mentors (n=2936 connections), Hispanic or Latinx mentees made up 22% (645/2936) of the accepted connections, non-Hispanic or Latinx mentees made up 72% (2121/2936), and those who identified as other were 6% (170/2936); see Figure 8. Non-Hispanic or Latinx mentors were a majority of the accepted connections (2389/2936, 81%), whereas Hispanic or Latinx mentors were 15% (429/2936) of the accepted connections. Of the accepted connections with Hispanic or Latinx mentees (n=645 connections), 64% (411/645) were with non-Hispanic or Latinx mentors and 30% (196/645) were with Hispanic or Latinx mentors. Of the accepted connections with Hispanic or Latinx mentors (n=429), 46% (196/429) were with Hispanic or Latinx mentees, 44% (189/429) with non-Hispanic or Latinx mentees, and 10% (44/429) with other ethnic backgrounds. Of the accepted connections with non-Hispanic or Latinx mentees (n = 2121), 88% (1862/2121) were with non-Hispanic or Latinx mentors, 9% (189/2121) with Hispanic or Latinx mentors, and 3% (70/2121) with other mentors. Of the accepted connections with non-Hispanic or Latinx mentors (n=2389), most were with non-Hispanic or Latinx mentees (1862/2389, 78%), followed by Hispanic or Latinx mentees (411/2389, 17%) and other mentees (116/2389, 5%). Accepted connections with mentees of other ethnicities occurred 170 times, and accepted connections with mentors of other ethnicities occurred 118 times.

#### Mentee-Mentee Accepted Connections by Ethnicity

**Figure 9:**
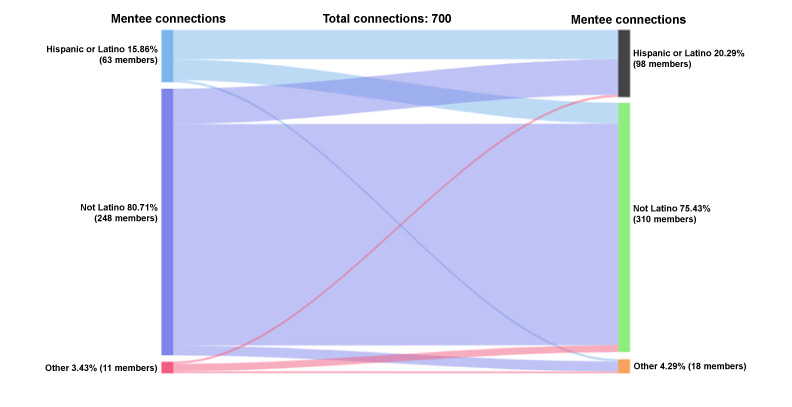
Connections by ethnicity: mentee-mentee.

Non-Hispanic or Latinx mentees represent 81% (565/700) of the initiated and accepted mentee-mentee connections, while Hispanic or Latinx mentees were 16% (111/700) of those connections (n=700 connections; Figure 9). Among the mentees who received and accepted peer mentee requests, 75% (528/700) were non-Hispanic or Latinx, 20% (142/700) were Hispanic or Latinx, and 4% (30/700) identified as other. Hispanic or Latinx mentees who initiated mentee-mentee connections (n=111 connections) reached out to Hispanic or Latinx (62/111, 56%), non-Hispanic or Latinx (44/111, 40%), and other (5/111, 5%) mentees. Non-Hispanic or Latinx mentees who initiated mentee-mentee connections (n=565) connected with non-Hispanic or Latinx (469/565, 83%), Hispanic or Latinx (75/565, 13%), and other (21/565, 4%) mentees. A total of 24 connections occurred for people of other ethnic backgrounds; one-third were with non-Hispanic or Latinx mentees.

#### Mentor-Mentor Accepted Connections by Ethnicity

**Figure 10:**
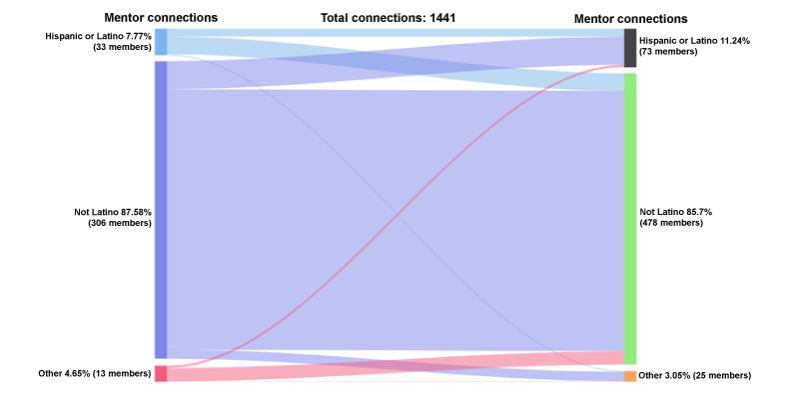
Connections by ethnicity: mentor-mentor.

Mentors can connect with each other, and of the mentors who initiated and accepted mentoring connections (n=1441 connections), 88% (1262/1441) were non-Hispanic or Latinx, 8% (112/1441) were Hispanic or Latinx, and 5% (67/1441) were other ethnicity (Figure 10). Among the mentors who received and accepted peer mentor requests, 86% (1235/1441) were non-Hispanic or Latinx, 11% (162/1441) were Hispanic or Latinx, and 3% (44/1441) were other ethnicity. Hispanic or Latinx mentors who initiated mentor-mentor connections (n=112 connections) reached out to non-Hispanic or Latinx (75/112, 67%), Hispanic or Latinx (33/112, 29%), and other (4/112, 4%) mentees. Non-Hispanic or Latinx mentors who initiated mentor-mentor connections (n=1262) connected with non-Hispanic or Latinx (1104/1262, 87%), Hispanic or Latinx (119/1262, 9%), and other (39/1262, 3%) mentors. A total of 67 connections occurred for people of other ethnic backgrounds; 84% (56/67) were with non-Hispanic or Latinx mentors.

### MSI-Carnegie Institutions

#### Mentee-Mentor Accepted Connections by the MSI-Carnegie Classification

**Figure 11:**
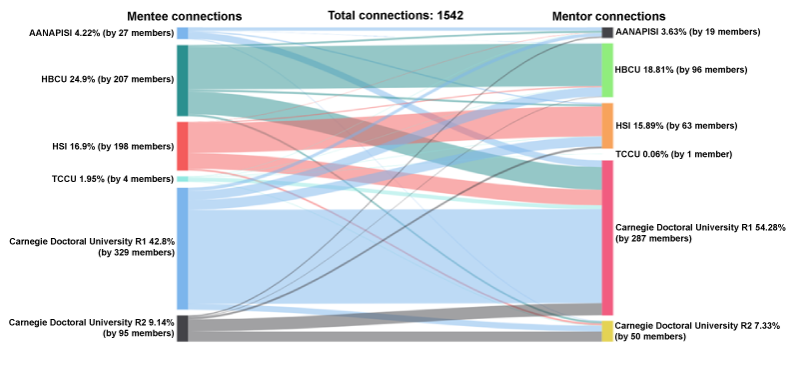
Connections by MSI Classification: mentee-mentor. AANAPISI: Asian American and Native American Pacific Islander–serving institution; HBCU: historically Black colleges and universities; HSI: Hispanic-serving institution; MSI: minority-serving institution; TCCU: tribally controlled colleges and universities.

Of the accepted connections between mentees and mentors with institution information (n=1542), mentees’ connections represented the following institutions: R1 (660/1542, 42%), HBCU (384/1543, 25%), HSI (262/1542, 17%), R2 (141/1542, 9%), AANAPISI (65/1542, 4%), and TCCU (30/1542, 2%); see Figure 11. Following a similar pattern, 54% (837/1542) of accepted mentor connections were at R1 universities, followed by 19% (290/1542) HBCUs, 16% (245/1542) HSIs, 7% (113/1542) R2 universities, 4% (56/1542) AANAPISIs, and less than 1% (1/1542) TCCUs (see Textbox 1 for a list of definitions for the MSI-Carnegie classification).

Of the 660 accepted connections from mentees at R1 universities, the mentors represented universities that were also R1 (50/660, 77%), followed by HSI (53/660, 8%), HBCU (49/660, 7%), R2 (32/660, 5%), and AANAPISI (18/660, 3%). Among the 384 accepted connections among mentees at HBCUs, a majority of mentors were from HBCUs (230/384, 60%) or R1 universities (122/384, 32%). The remaining mentors came from other university designation types for less than 3%. There were 262 accepted connections for mentees from HSIs. Most of the connections were with other HSIs (161/262, 61%) and R1 universities (84/262, 32%), followed by R2 universities (10/262, 4%) and HBCUs (6/262, 2%). Of the 141 accepted connections for mentees at R2 universities, almost half connected with R1 mentors (66/141, 47%) and over a third with R2 mentors (54/141, 38%). The remaining mentors came from HSIs (11/141, 8%), AANAPISIs (7/141, 5%), and HBCUs (3/141, 2%). A total of 65 connections were accepted for mentees from AANAPISIs, who connected with mentors from R1 universities (36/65, 55%), AANAPISIs (18/65, 28%), HSIs (7/65, 11%), R2 universities (3/65, 5%), and HBCUs (1/65, 2%). Only 30 connections were accepted for mentees from TCCUs; 70% (21/30) of those connections were with R1 university mentors.

**Minority-serving institution–Carnegie classification list.**

- AANAPISI: Asian American and Native American Pacific Islander serving institution
- HBCU: historically black colleges and universities
- HIS: Hispanic-serving institution
- TCCU: tribal college or university
- Carnegie doctoral university R1: doctoral universities—very high research activity
- Carnegie doctoral university R2: doctoral universities—high research activity

Of the 837 connections accepted by R1 university mentors, mentees were from institutions designated as R1 (508/837, 61%), HBCU (122/837, 15%), HSI (84/837, 10%), R2 (66/837, 8%), AANAPISI (36/837, 4%), and TCCU (21/837, 3%). A total of 290 connections were accepted by HBCU mentors, with mentees from HBCUs (230/290, 79%), R1 universities (49/290, 17%), HSIs (6/290, 2%) and R2 universities (3/290, 1%). Of the 245 connections accepted by HSI mentors, mentees represented HSIs (161/245, 66%), R1 (53/245, 22%) and R2 (11/245, 4%) universities, HBCUs (11/245, 4%), AANAPISIs (7/245, 3%), and TCCUs (2/245, 1%). A total of 113 connections were accepted by mentors at R2 universities who connected with mentees from R2 universities (54/113, 48%), R1 universities (32/113, 28%), HBCUs (11/113, 10%), HSIs (10/113, 9%), AANAPISIs (3/113, 3%), and TCCUs (3/113, 3%). Of the 56 connections accepted by mentors at AANAPISIs, one-third were with mentees from AANAPISIs and R1 universities (18/56 each). The remaining came from HBCUs (10/56, 18%), R2 universities (7/56, 13%), TCCUs (2/56, 4%), and HSIs (1/56, 2%). Only 1 connection was accepted by a mentor at a TCCU.

#### Mentee-Mentee Accepted Connections by the MSI-Carnegie Classification

**Figure 12:**
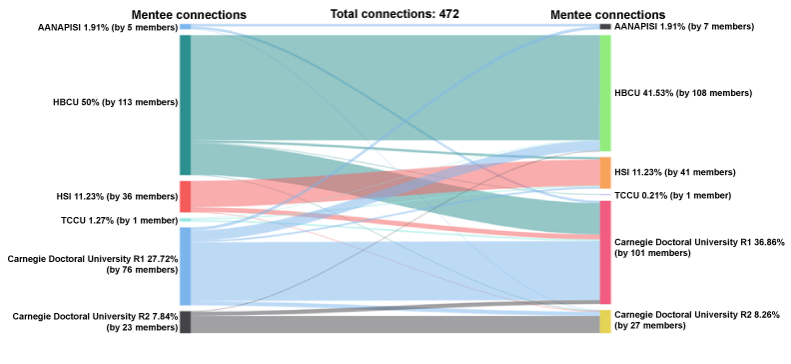
Connections by MSI Classification: mentee-mentee. AANAPISI: Asian American and Native American Pacific Islander–serving institution; HBCU: historically Black colleges and universities; HSI: Hispanic-serving institution; MSI: minority-serving institution; TCCU: tribally controlled colleges and universities.

HBCU mentees represent 50% (236/472) of initiated and accepted mentee-mentee connections (n=472 connections), followed by 28% (131/472) R1 mentees, 11% (53/472) HSI mentees, 8% (37/472) R2 mentees, 2% (9/472) AANAPISI mentees, and 1% (6/472) TCCU mentees (Figure 12). Among the mentees who received and accepted peer mentee requests, 42% (196/472) were from HBCUs, 37% (174/472) were from R1 universities, 11% (53/472) were from HSIs, 8% (39/472) were from R2 universities, 2% (9/472) were from AANAPISIs, and less than 1% (1/472) were from TCCUs.

HBCU mentees who initiated peer mentee-mentee connections (n=236 connections) connected with mentees from predominantly HBCUs (177/236, 75%), followed by R1 universities (53/236, 22%) and only 2% (4/236) HSIs. R1 mentees who initiated peer mentee-mentee connections (n=131 connections) connected with mentees from R1 universities (99/131, 76%), HBCUs (17/131, 13%), R2 universities (7/131, 5%), AANAPISIs (5/131, 4%), and HSIs (3/131, 2%). HSI mentees who initiated peer mentee-mentee connections (n=53 connections) connected with mentees from HSIs (44/53, 83%), R1 universities (8/53, 15%), and R2 universities (1/53, 2%). R2 mentees who initiated peer mentee-mentee connections (n=37) connected with R2 universities (29/37, 78%), R1 universities (7/37, 19%), and HBCUs (1/37, 3%). TCCUs and AANAPISIs had fewer than 10 connections each.

#### Mentor-Mentor Accepted Connections by MSI Designation

**Figure 13:**
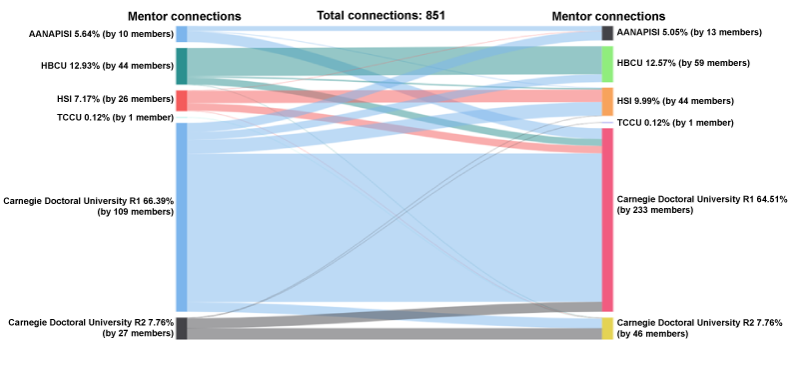
Connections by MSI Classification: mentor-mentor. AANAPISI: Asian American and Native American Pacific Islander–serving institution; HBCU: historically Black colleges and universities; HSI: Hispanic-serving institution; MSI: minority-serving institution; TCCU: tribally controlled colleges and universities.

Of the mentors who initiated and accepted mentoring connections (n=851 connections), 66% (565/851) were R1 connections followed by 13% (110/851) HBCUs, 8% (66/851) R2 universities, 7% (61/851) HSIs, 6% (48/851) AANAPISIs, and less than 1% (1/851) TCCUs (Figure 13). Among the mentors who received and accepted peer mentor requests, 65% (549/851) were from R1 institutions, 13% (107/851) from HBCU institutions, 10% (85/851) from HSIs, 8% (66/851) from R2 institutions, 5% (43/851) from AANAPISIs, and less than 1% (1/851) from TCCU institutions.

Among the 565 connections initiated by mentors at R1 universities, connections were with mentors at other R1 universities (444/565, 79%), followed by HSIs (41/565, 7%), AANAPISIs (28/565, 5%), R2 universities (29/565, 5%), and HBCUs (23/565, 4%). Among the connections sent by mentors at HBCUs (110 connections), 76% (84/110) were with mentors at other HBCUs, 19% (21/110) to R1 institutions, HSIs (4/110, 4%), and R2 universities (1/110, 1%). R2 mentors connected (n=66 connections) with predominantly R2 (34/66, 52%) or R1 (30/66, 45%) institutions, and only 1 connection each for HSIs and TCCUs. Mentors from HSI locations connected (n=61 connection) with mostly mentors from HSIs (37/61, 61%), followed by R1 universities (22/61, 36%) and only 1 connection each for AANAPISIs and R2 universities. Mentors from AANAPISIs connected (n=48 connections) with R1 mentors (32/48, 67%) and AANAPISI mentors (14/48, 29%). Only 2 connections were made with HSI mentors. Only 1 mentor connection was initiated from a mentor at a TCCU.

### Educational Attainment

#### Mentee-Mentor Accepted Connections by Education Level

**Figure 14:**
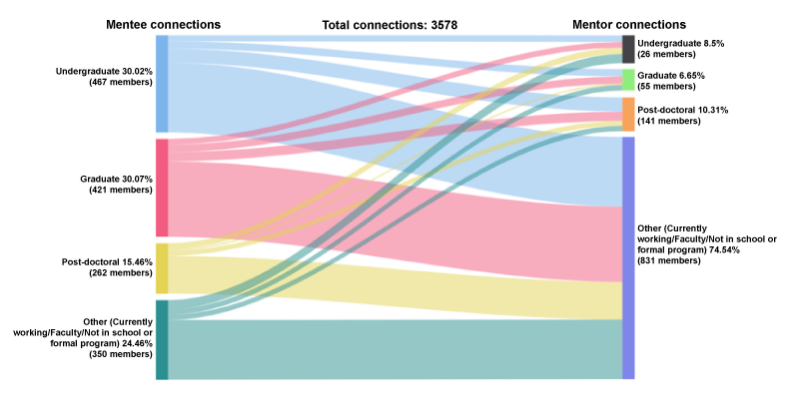
Connections by educational attainment: mentee-mentor.

Of the accepted connections between mentees and mentors with educational attainment data (n=3578), mentees have the following distribution for education level: undergraduate (1074/3578, 30%), graduate (1076/3578, 30%), postdoctoral fellow (553/3578, 15%), and other (ie, currently working, faculty, etc; 875/3578, 24%); see Figure 14. In contrast, most mentor-accepted connections were with mentors working in the field (2667/3578, 75%), followed by postdocs (369/3578, 10%), undergraduate students (304/3578, 9%), and graduate students (238/3578, 7%).

Of the 1074 accepted connections from undergraduate mentees, the mentors were currently working (767/1074, 71%), postdocs (155/1074, 14%), graduate students (81/1074, 8%), and other undergraduate students (71/1074, 7%). Among the 1076 accepted connections from graduate mentees, the mentors were currently working (829/1076, 77%), postdocs (102/1076, 10%), graduate students (81/1076, 8%), and undergraduate students (64/1076, 6%). Fewer mentee connections occurred from people currently working (n=875). They connected with people who were also currently working (657/875, 75%), undergraduate students (100/875, 11%), graduate students (60/875, 7%), and postdocs (58/875, 7%). There were 553 connections from postdocs connecting to people currently working (414/553, 75%), undergraduate students (69/553, 12%), other postdocs (54/553, 10%), and graduate students (16/553, 3%).

Of the 2667 connections accepted by mentors currently working, mentees were graduate students (829/2667, 31%), undergraduate students (767/2667, 29%), others currently working (657/2667, 25%), and postdocs (414/2667, 16%). A similar number of connections were accepted by postdoctoral (n=369 connections) and undergraduate (n=304) mentors. Connections with postdoctoral mentors came from undergraduate (155/369, 42%), graduate school (102/369, 28%), currently working (58/369, 16%), and other postdoctoral (54/369, 15%) mentees. Connections with undergraduate mentors came from working (100/304, 33%), postdoctoral or undergraduate (71/304, 23%), and graduate student (64/304, 21%) mentees. A total of 238 connections were accepted by graduate student mentors from undergraduate (81/238, 34%) and graduate students (81/238, 34%), people currently working (60/238, 25%), and postdocs (16/238, 7%).

#### Mentee-Mentee Accepted Connections by Educational Attainment

**Figure 15:**
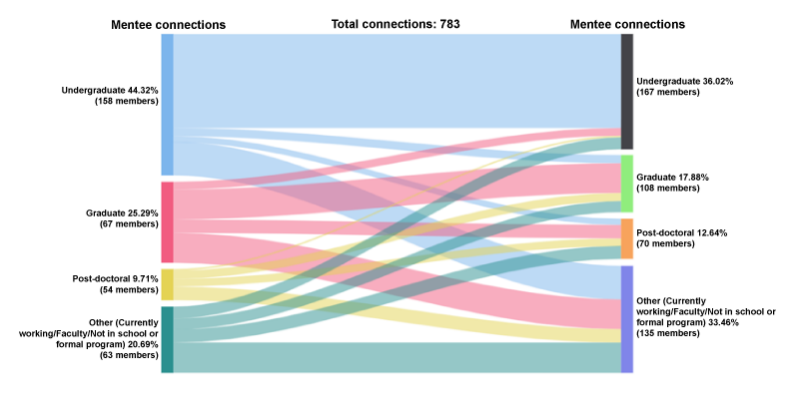
Connections by educational attainment: mentee-mentee.

Undergraduate students represent 44% (347/783) of initiated and accepted mentee-mentee connections (n=738 connections), followed by graduate students (198/783, 25%), people currently working (162/783, 21%), and postdocs (76/783, 10%); see Figure 15. Among the mentees who received and accepted peer mentee requests, 26% (282/783) were undergraduate students, 33% (262/783) were people working in the field, 18% (140/783) were graduate students, and 13% (99/783) were postdocs.

Undergraduate mentees who initiated peer mentee-mentee connections (n=347 connections) connected with undergraduate mentees (230/347, 66%), people working (82/347, 24%), graduate mentees (20/347, 6%), and postdocs (15/347, 4%). Among the 198 connections with graduate mentees, 37% (73/198) were with mentees who were also graduate students, people currently working (73/198, 37%), postdocs (33/198, 17%), and undergraduates (19/198, 6%). Currently working mentees had 162 peer connections with mainly other people working (74/162, 46%), followed by postdocs (32/162, 20%), undergraduate mentees (29/162, 18%), and graduate mentees (27/162, 17%). Postdoc mentees initiated 76 connections with people currently working (33/76, 43%), graduate students (20/76, 26%), other postdocs (19/76, 25%), and undergraduate students (4/76, 5%).

#### Mentor-Mentor Accepted Connections by Educational Attainment

**Figure 16:**
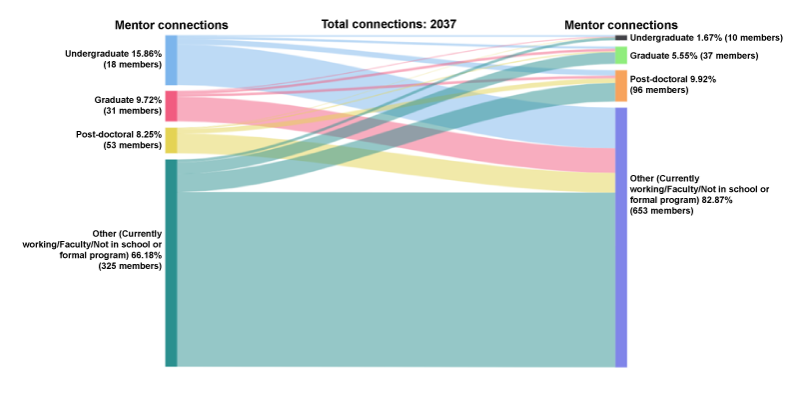
Connections by educational attainment: mentor-mentor.

Of the mentors who initiated and accepted mentoring connections (n=2037 connections), 66% (1348/2037) were with people currently working, followed by undergraduates (323/2037, 16%), graduate students (198/2037, 10%), and postdocs (168/2037, 8%); see Figure 16. Among mentors who received and accepted peer mentor requests, a majority were from people currently working (1688/2037, 83%) followed by postdocs (202/2037, 10%), graduate students (113/2037, 6%), and undergraduate students (34/2037, 2%).

Among the 1348 connections initiated by currently working mentors, connections were with mentors currently working (1136/1348, 84%), postdocs (119/1348, 9%), graduate students (75/1348, 6%), and undergraduate students (18/1348, 1%). Undergraduate mentors initiated 323 connections with mentors currently working (264/323, 82%), postdocs (35/323, 11%), graduate students (15/323, 5%), and undergraduates (9/323, 3%). Within connections from graduate student mentors (198 connections), most were also to people currently working (160/198, 81%), followed by postdocs (17/198, 9%), graduate students (16/198, 8%), and undergraduates (5/198, 3%). Among the connections sent by postdoc mentors (168 connections), most were with either people currently working (128/168, 76%) or other postdocs (31/168, 18%).

## Discussion

### Principal Findings

Mentoring is a vital aspect of training to create the next generation of a diverse biomedical research workforce. The MyNRMN platform provides a powerful tool to enable remote mentoring across institutions throughout the United States and territories. Before this study, the utility of the mentoring connections and mentoring networks across institutions and how mentors and mentees connected was unknown. This study aimed to examine the large-scale mentoring connections facilitated by our web-based platform between students (mentees) and faculty (mentors) across institutional and geographic boundaries by gender, race, ethnicity, institution type, and educational attainment.

We developed the MyNRMN platform to increase access to diverse mentoring for mentees across institutions and geographic boundaries across the United States and territories. Access to a diverse and more extensive mentoring network grows the social capital of the mentees. It was hypothesized that by providing a remote platform accessible to a large swath of biomedical students, as well as individuals in the workforce, mentoring connections would occur [7]. Our previous work described the intentional recruitment strategies across the United States, with a specific emphasis on minority-serving institutions and conferences that reach diverse audiences [5]. The results of these successful strategies provide evidence demonstrating that the hypothesis is correct, as observed by the substantial number of connections between heterogeneous individuals. As such, the MyNRMN platform is addressing the NIH’s initiative to provide mentoring support to underrepresented students and scientists [2,18]. By addressing this initiative, we can now observe the growth of individuals’ social capital, which is crucial to their persistence and advancement in the biomedical sciences [7].

As evident from the data in this study, the MyNRMN platform provides a diverse cohort of mentees access to diverse mentors across the nation. Our platform’s goal is to increase diversity of the biomedical workforce, and our mentees are predominantly Black compared with other racial groups, representing a key demographic of interest by the NIH [19]. Other racial and ethnic groups also have strong representation on the MyNRMN platform, and there are opportunities to further expand in some subgroups, such as Hispanic or Latinx students. Previous research found that Latina women in the biomedical sciences experience isolation and a low sense of belonging in their undergraduate programs [20]; thus remote mentoring and connections may bridge the gap for less inclusive environments in biomedical science programs. Additionally, we found that a majority of mentees and mentors on this platform identified as female. In fact, there was a gender skew in the proportion of requested and accepted connections to female mentors. This finding may represent a mentoring burden that women, especially women of color, face in academia and training [21]. Future work could assess the MyNRMN mentors’ perceived workload and burden in these mentoring roles to determine how to ease any encumbrance through the platform. It would also be imperative to assess the variability of workloads across demographics and identities, such as race and ethnicity, and gender, respectively.

Another benefit of the MyNRMN platform is the connectivity with mentors at other institutions. This ability to connect beyond institutional boundaries is crucial for Black or African American and Hispanic or Latinx individuals as it provides support and builds a community of mentors enabling persistence, providing role models, and increasing social capital in spaces that were previously untapped or underresourced [1]. There were over 4500 connections between mentors and mentees at different institutions, including significant crossover among HBCUs, MSIs, and R1 and R2 institutions. These connections are contingent on a bridge due to location, resources, and physical distance. An added benefit of these connections is that some institutions may not have senior faculty members with the bandwidth for mentoring or even a faculty member within a mentee’s specific discipline. Additionally, remote mentoring can help foster conversations that may not occur in an in-person environment as demonstrated by mentoring during the COVID-19 pandemic [22]. Furthermore, having a mentor outside of a person’s immediate proximity can provide valuable insight on professional development [23] that cannot be obtained within the organizational culture of a home institution. MyNRMN provides a solution to enable these cross-institutional and long-distance collaborations. Future research should evaluate the ability to recruit and sustain these cross-institutional partnerships on the web-based platform.

The data reported represent connections as the unit of observation rather than persons; thus, some persons may have more connections than others and be more heavily represented in the data. We provided the average number of connections in Table 1 to demonstrate these potential differences by demographic. Additionally, data on demographic characteristics are passively collected based on profile forms on the network, and thus, missing data are an area of concern. As of now, we do not actively collect many elements of diversity, including LGBTQIA+ identity, which is an important facet of the lived experience [24]. Thus, the findings from this paper should be considered in the context of potential limitations. In the future, we plan to adjust our data profile fields to be representative of sexual and gender underrepresented groups. Moreover, we did not present data on nonaccepted connections, meaning a user reached out to another user to connect, but the connection was not accepted. It should be noted that all mentor and mentee participation on the platform is voluntary, and competing demands may result in a nonacceptance. We have also observed that connection acceptances are cyclical with the ebbs and flows of the academic semesters when workloads may shift. A mentor’s participation is also conditional based on their capacity to engage with additional mentees outside of any requirements or obligations for their position. Future research will explore user experiences with the platform to inform adjustments to meet end-user needs.

### Conclusions

Access to mentors is crucial for career advancement and increasing our nation’s current and future biomedical workforce. We developed a web-based national platform to connect mentees and mentors across institutions and geographic boundaries toward this goal. The MyNRMN platform is achieving this goal by facilitating mentoring connections and developing diverse mentoring networks for diverse mentees. By analyzing the organic evolution of mentoring connections throughout MyNRMN, we can observe the value of facilitating and nurturing these connections. In this study, we examined large-scale mentoring connections and the diversity of these connections and addressed a gap in our understanding of how mentees and mentors connect across institutions throughout the United States. We observed that a web-based remote space for mentors and mentees to connect and build their network can enable diverse connections between mentors and mentees.

## Abbreviations

AANAPISI: Asian American and Native American Pacific Islander–serving institution
HBCU: historically Black colleges and universities
HSI: Hispanic-serving institution
MSI: minority-serving institution
NIH: National Institutes of Health
NRMN: National Research Mentoring Network
TCCU: tribally controlled colleges and universities
